# Facile synthesis of an acid-responsive cinnamaldehyde-pendant polycarbonate for enhancing the anticancer efficacy of etoposide *via* glutathione depletion†

**DOI:** 10.1039/d4ra02468k

**Published:** 2024-05-13

**Authors:** Shaojie Wu, Kuofei Liao, Jiamin Chen, Feng Li

**Affiliations:** a Key Laboratory of Biomedical Polymers of Ministry of Education, College of Chemistry and Molecular Science, Wuhan University Wuhan 430072 PR China lifeng@whu.edu.cn

## Abstract

Glutathione (GSH) is an important antioxidant that maintains cellular redox homeostasis and significantly contributes to resistance against various chemotherapeutic agents. To address the challenge of GSH-mediated drug resistance in etoposide (ETS), we developed a facile synthetic method to prepare a biocompatible acid-responsive polycarbonate (PEG-PCA) containing cinnamaldehyde (CA), a potent GSH-depleting agent, as a side chain using nontoxic raw materials. This polymer self-assembled in aqueous solutions to form nanoparticles (ETS@PCA) that encapsulated ETS, enhancing its water solubility and enabling tumor-targeted delivery. *In vitro* studies demonstrated that ETS@PCA could respond to the acidic tumor microenvironment, releasing CA to rapidly deplete GSH levels. Consequently, ETS@PCA exhibited superior cytotoxicity compared to free ETS. Furthermore, *in vivo* experiments corroborated the enhanced tumor inhibitory effects of ETS@PCA.

## Abstract 2

A facile synthetic method was developed to prepare a biocompatible acid-responsive polycarbonate containing cinnamaldehyde moieties, a potent GSH-depleting agent, for enhancing the anticancer efficacy of etoposide.

## Graphical abstract

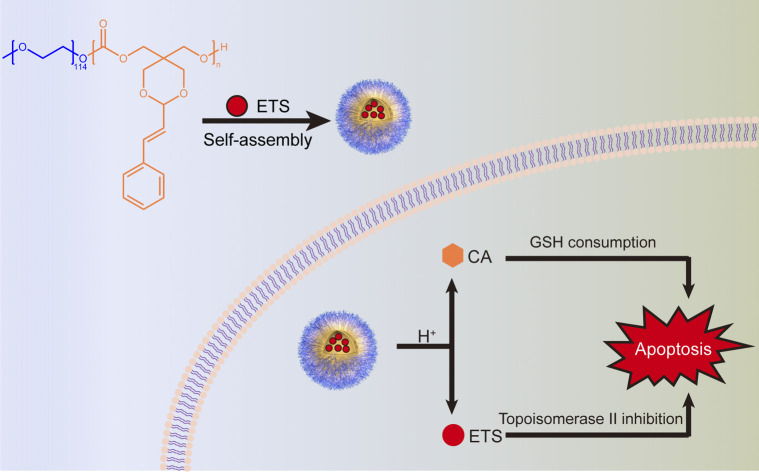

## Introduction

Despite significant strides in drug discovery and our growing understanding of cancer biology, achieving complete cancer treatment remains a formidable challenge.^1^ Conventional cancer therapies, such as chemotherapy, are often limited by their inability to distinguish between cancerous and healthy cells, resulting in debilitating side effects for many patients.^2^ A fundamental goal in cancer therapy is the selective targeting and elimination of malignant cells. Researchers have identified several significant features in tumor microenvironment (TME) that differ from normal tissue, including weakly acidic pH, hypoxia, and increased levels of glutathione (GSH). This combination of properties empowers cancer cells to proliferate at an accelerated rate and develop resistance to certain conventional therapeutic modalities.^3^ Consequently, exploiting these disparities between TME and healthy tissues presents a promising avenue for novel cancer therapies. GSH is a tripeptide composed of glutamate, cysteine, and glycine. It plays a pivotal role in detoxification of carcinogens, protection against oxidative stress, and modulation of intracellular and intercellular signaling pathways.^4^ Being the most abundant non-protein thiol in cells, GSH is a potent nucleophile that readily reacts with xenobiotic electrophiles, thereby facilitating tumorigenesis, progression, and metastasis.^5,6^ Since GSH confers resistance to diverse cancer therapies, depleting GSH in tumor cells emerges as a persuasive strategy to enhance treatment efficacy and promote tumor cell death.^7^

Cinnamaldehyde (CA), a natural compound abundant in plants such as cinnamon, has been found to be effective in reducing intracellular GSH levels.^8^ This is attributed to the α, β-unsaturated aldehyde structure of CA, which readily reacts with sulfhydryl groups, the primary physiologically active source of GSH.^9^ Researchers have successfully employed CA to deplete GSH in various cancer therapeutic regimens, including reducing glutathione detoxification to enhance chemotherapeutic agent efficacy,^10^ decreasing reactive oxygen species (ROS) scavenging to amplify the effects of ROS-based therapies,^11^ and inducing non-apoptotic cell death through ferroptosis.^12^ For instance, Zhu *et al.* developed a GSH-responsive mesoporous organosilicon nanoparticle nanomedicine, MON-CA-TPP@HA. Upon the cleavage of MON disulfide bonds triggered by overexpression of GSH in cancer cells, CA can be released to induce GSH consumption and activate oxidative stress, thereby inducing apoptosis and immunogenic cell death (ICD) in breast cancer cells.^13^

The highly reactive aldehyde group in CA renders it susceptible to rapid oxidation in the bloodstream. However, despite extensive research on CA as a potential chemotherapeutic agent, its low bioavailability impedes direct clinical application. Therefore, the presence of the aldehyde functional group also allows for facile chemical modification of CA, facilitating their subsequent assembly into stimuli-responsive drug delivery systems, thereby improving the bioavailability of CA.^14–16^ Owing to the remarkable structural diversity and tunability of aliphatic polycarbonates,^17^ a CA-functionalized aliphatic polycarbonate (PEG-PCA) was developed as a drug carrier in this study. CA segments were incorporated into the side chain through acid-cleavable linkages to mitigate the detrimental effects of GSH on anticancer drugs. Exploiting the weakly acidic pH prevalent in tumors and intracellular compartments, such as endosomes and lysosomes, CA-containing nanomedicines can be triggered for release and accumulate at targeted sites.^18^ Additionally, the aliphatic polycarbonates can undergo biodegradation and are subsequently excreted from the body after fulfilling their function within the organism.^19^

**Scheme 1:**
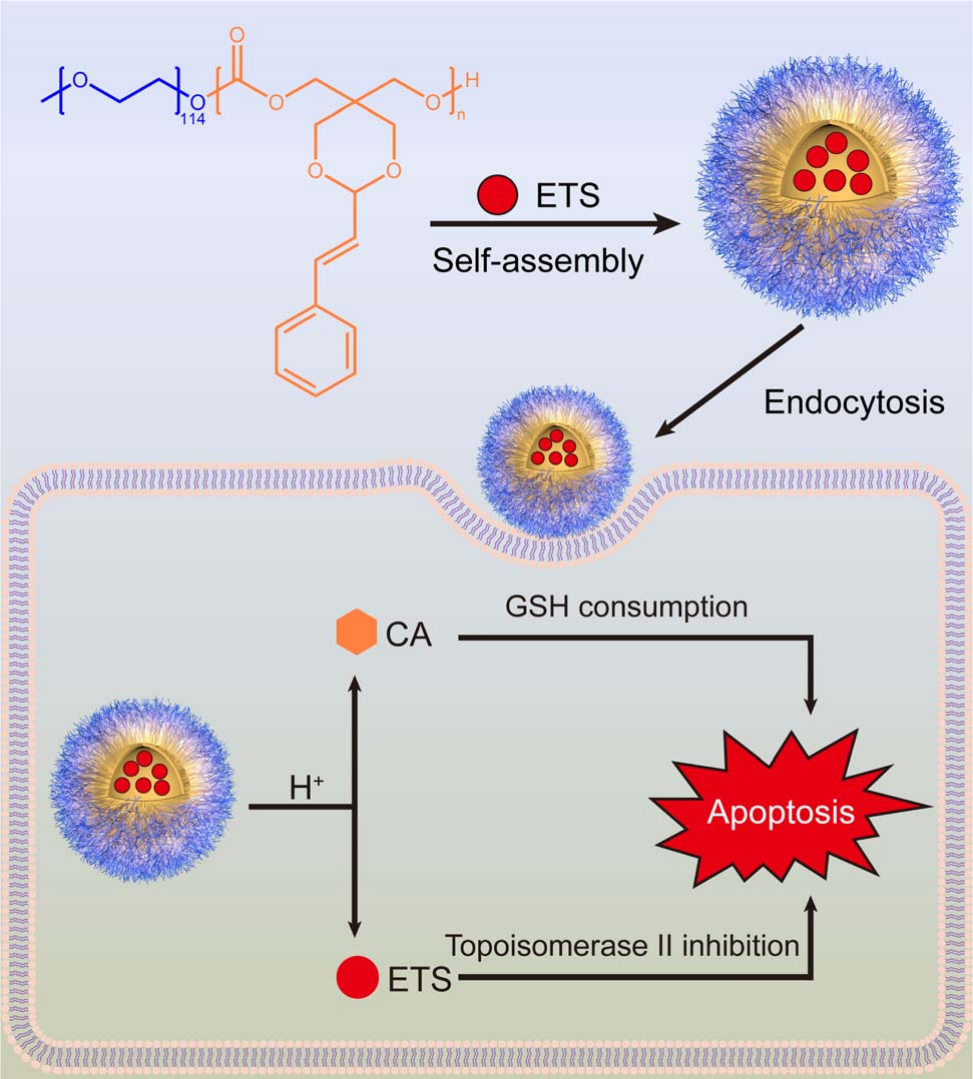
Synthesis and apoptosis mechanism of ETS@PCA nanomedicine.

Adhering to the principles of minimizing side effects and promoting biodegradability, a CA-containing cyclic carbonate monomer was synthesized using a nontoxic reactant pentaerythritol *via* a straightforward two-step reaction. The monomer was subsequently polymerized through a ring-opening reaction to yield the final block copolymer, methoxy polyethylene glycol-*b*-polycarbonate-*g*-cinnamaldehyde (PEG-PCA). The amphiphilic PEG-PCA can encapsulate the anticancer drug etoposide (ETS) within the hydrophobic core, forming nano-micelles that facilitate controlled drug release. ETS is a well-established chemotherapeutic agent used to treat various cancers, such as neuroblastoma, lymphoma, testicular cancer, and lung cancer.^20^ However, its efficacy is hindered by GSH and GSH-related reactions.^21^ Therefore, we aim to enhance the efficacy of ETS through CA while improving its water solubility and bioavailability by encapsulating it in polymeric nanoparticles. As shown in Scheme 1, upon internalization, the drug carriers encounter a pH gradient within the endocytic vesicles, triggering the hydrolysis of the acetal linkage on the hydrophobic segment that links the CA moiety, increasing the hydrophilicity and degradation rate of the polycarbonate backbone, facilitating the release of CA and ETS into the cytosol of target cancer cells, where CA exerts a synergistic effect on alleviating the deficiency of ETS by consuming GSH.

## Experimental

### Materials

Cinnamaldehyde (CA), mPEG_5k_ and triphosgene (BTC) were purchased from Macklin Biochemical Co., Ltd (Shanghai, China). Etoposide and 1,8-diazabicyclo[5.4.0]undec-7-ene (DBU) were obtained from TCI Co., Ltd (Shanghai, China). All other chemicals included in organic synthesis were provided by Guoyao Chemical Reagent Co. Ltd (Shanghai, China). Tetrahydrofuran (THF) and triethylamine (TEA) were freshly distilled from CaH_2_ before use. Unless noted, all commercial reagents were used as received without further purification.

Roswell Park Memorial Institute (RPMI) 1640 medium, penicillin-streptomycin solution and fetal bovine serum (FBS) were purchased from XP Biomed Ltd (Shanghai, China). Cell Counting Kit-8 (CCK8) was bought from Meilun Biotech Co., Ltd (Dalian, China). Reduced Glutathione Content Assay Kit and Reactive Oxygen Species Assay Kit were obtained from Solarbio Science & Technology Co., Ltd (Beijing, China). Annexin V-FITC/PI Apoptosis Kit was purchased from Procell Life Science & Technology Co., Ltd (Wuhan, China).

### Synthesis of (2-styryl-1,3-dioxane-5,5-diyl) dimethanol (PERCA)

PERCA was synthesized following a reported procedure.^22^ Pentaerythritol (25.0 g, 184 mmol) was dissolved in hot water (180 mL) and then the mixture was allowed to cool to room temperature undisturbed. Concentrated hydrochloric acid (950 μL) and CA (1.0 mL, 7.9 mmol) were added to the solution being stirred in sequence. Upon precipitate formation, an additional 22.5 mL of CA (179 mmol) was added dropwise, and the resulting reaction mixture was stirred at room temperature for 4 hours. The crude product was isolated by filtration, washed with ice-cold Na_2_CO_3_ solution and ethyl ether, and dried under vacuum to yield a white solid. (33.6 g, 73%) ^1^H NMR (400 MHz, DMSO-*d*_6_) *δ* (ppm): 7.49–7.26 (m, 5H), 6.72 (d, *J* = 16.2 Hz, 1H), 6.20 (dd, *J* = 16.2, 4.6 Hz, 1H), 5.03 (d, *J* = 4.6 Hz, 1H), 4.59 (t, *J* = 5.4 Hz, 1H), 4.51 (t, *J* = 5.1 Hz, 1H), 3.84 (d, *J* = 11.6 Hz, 2H), 3.68 (d, *J* = 11.6 Hz, 2H), 3.60 (d, *J* = 5.3 Hz, 2H), 3.21 (d, *J* = 5.3 Hz, 2H). ^13^C NMR (400 MHz, DMSO-*d*_6_) *δ* (ppm): 136.24, 132.64, 129.14, 128.63, 127.12, 126.77, 100.44, 69.13, 61.50, 59.95, 39.52. ESI-MS *m/z* [M + H]^+^ = 251.13.

### Synthesis of 9-styryl-2,4,8,10-tetraoxaspiro[5.5]undecan-3-one (STO)

A mixture of PERCA (4.80 g, 19.2 mmol), THF (300 mL) and TEA (10.0 mL) was charged into an oven-dried flask under argon. The solution was cooled to −78 °C, followed by the dropwise addition of a solution of BTC (2.90 g, 9.77 mmol) in THF (60 mL). After the addition was complete, the reaction mixture was stirred at ambient temperature for 3 hours. The precipitated triethylammonium chloride salt was filtered off, and the filtrate was concentrated under reduced pressure. Subsequently, the residue was redissolved in CH_2_Cl_2_ (250 mL), washed with 5% NaHCO_3_ (3 × 100 mL), dried over Na_2_SO_4_ and evaporated to afford the crude product, which was further purified by recrystallization from THF/Et_2_O to give a white solid. (2.97 g, 56%) ^1^H NMR (400 MHz, CDCl_3_) *δ* (ppm): 7.42–7.27 (m, 5H), 6.79 (d, *J* = 16.2 Hz, 1H), 6.17 (dd, *J* = 16.2, 4.6 Hz, 1H), 5.13 (d, *J* = 4.6 Hz, 1H), 4.65 (s, 2H), 4.13 (t, *J* = 12.0 Hz, 2H), 4.05 (s, 2H), 3.77 (t, *J* = 12.0 Hz, 2H). ^13^C NMR (400 MHz, CDCl_3_) *δ* (ppm): 147.90, 135.53, 134.43, 128.67, 128.60, 126.91, 123.94, 101.66, 71.37, 70.31, 69.07, 31.60. ESI-MS *m/z* [M + H]^+^ = 277.11.

### Synthesis of amphiphilic block copolymer (PEG-PCA)

PEG-PCA was prepared through STO polymerization initiated by mPEG, using DBU/TU as the catalyst. In a typical experiment, mPEG_5k_ (0.80 g, 0.20 mmol) and STO (0.66 g, 2.4 mmol) were dissolved in anhydrous CH_2_Cl_2_ (6.0 mL) in a Schlenk flask fitted with a stirrer bar. Following three freeze–pump–thaw cycles, TU (88 mg, 0.24 mmol) and DBU (30 μL, 0.20 mmol) were added to catalyze the ring-opening polymerization. The reaction vessel was sealed and placed in an oil bath at 30 °C for 96 hours. The reaction mixture was dialyzed successively against CH_2_Cl_2_, DMSO, and ultrapure water. The final product was obtained as a white powder after lyophilization. ^1^H NMR (400 MHz, CDCl_3_): *δ* = 7.42–7.26 (m, ArH), 6.77 (d, CHCHPh), 6.17 (dd, CHCHPh), 5.08 (s, CH-acetal), 4.52 (s, –COCH_2_C–), 4.09 (s, –OCH_2_CCH_2_O–), 4.00 (s, –COCH_2_C–), 3.79 (s, –OCH_2_CCH_2_O–), 3.64 (s, –CH_2_OCH_2_–), 3.38 (s, PEG-OCH_3_). GPC data: *M*_n_ = 8500, *M*_w_/*M*_n_ = 1.27.

### Measurement of critical micelle concentration (CMC)

The CMC value was determined using pyrene as a fluorescence probe. Aqueous solutions of PEG-PCA were prepared at varying concentrations (0.0001–1.0 mg mL^−1^). A trace amount of pyrene in acetone was added to achieve a final pyrene concentration of 6 × 10^−7^ M. The acetone was removed completely by shaking the solutions for 8 hours in a 25 °C water bath. Fluorescence emission spectra of the polymer solutions were recorded from 350 to 450 nm with an excitation wavelength of 334 nm using a Shimadzu RF-5301PC Spectrofluorophotometer. The intensity ratio (*I*_373_/*I*_384_) from the pyrene emission spectra was monitored as a function of polymer concentration. The CMC value is defined as the polymer concentration at the inflection point of this curve.

### Preparation and characterization of polymeric nanoparticles

ETS-loaded PEG-PCA nanoparticles (ETS@PCA) were prepared according to the dialysis method under dark conditions. In brief, 50 mg of PEG-PCA and 5 mg of ETS were dissolved in 5 mL of DMSO and then added dropwise to 45 mL of phosphate-buffered saline (PBS, pH 7.4) under sonication. The solution underwent dialysis against ultrapure water for 20 hours, followed by filtration through a 0.22 μm syringe filter and lyophilization to obtain a white powder. Blank PEG-PCA nanoparticles (PCA) and C6-loaded nanoparticles (C6@PCA) were prepared using the same method. The drug loading content (DL%) and encapsulation efficiency (EE%) of ETS were determined by high-performance liquid chromatography (HPLC, Waters e2695).

### *In vitro* drug release

The release of the drug from ETS@PCA was studied in PBS (pH = 5.0, 6.8, 7.4). ETS@PCA (containing 0.5 mg CA, dissolved in 4 mL buffer) was added into a dialysis bag (*M*_W_CO = 3500 Da) and then immersed in 36 mL of phosphate buffer shaking (100 rpm) at 37 °C. At predetermined time intervals, 3.0 mL of the release medium was withdrawn from each tube and replaced with an equal volume of fresh buffer at the same temperature and pH. The drug concentration of the samples was analyzed by HPLC.

### Cell uptake

4T1 cell line was used to assess the *in vitro* cell experiments, which was purchased from Procell Life Science & Technology and maintained in RPMI 1640 supplemented with 10% FBS.

The cellular internalization of the micelles was investigated by using C6 as a fluorescent probe (C6: 250 ng mL^−1^). Briefly, 4T1 cells were seeded in a confocal dish at a density of 1 × 10^5^ cells per well and incubated at 37 °C for 24 h. Then, the cells were exposed to a fresh medium containing C6@PCA for 1 h, 3 h, or 5 h. Subsequently, DAPI and LysoTracker Red were added and incubated for 20 min. After washing with PBS three times, the cells were imaged by a confocal laser scanning microscope (CLSM).

### *In vitro* consumption of GSH and evaluation of ROS

To measure the GSH content, 4T1 cells were seeded at a density of 2 × 10^5^ cells per well in a 6-well plate and cultured for 24 hours to allow cell adhesion. Following a 24 hours treatment with PCA, ETS, and ETS@PCA (ETS concentration: 2 μM), cells were harvested and washed twice with PBS. Cell lysis was performed using ultrasonication on ice. The resulting lysate was centrifuged at 8000×*g* for 10 minutes, and the supernatant was used to measure GSH content using assay kits according to the manufacturer's instructions. Intracellular ROS levels were quantified by staining cells with the fluorescent probe 2′, 7′-dichlorofluorescein diacetate (DCFH-DA) after treating 4T1 cells with PCA, ETS, and ETS@PCA at equivalent concentrations. Fluorescence intensity was then measured using flow cytometry. Untreated control cells were assigned 100% for both GSH and ROS levels for normalization. All experiments were performed in triplicate.

### Cytotoxicity analysis

CCK-8 assay against 4T1 cells was carried out to detect cell viability by the manufacturer's instructions. Briefly, 4T1 cells were distributed in a 96-well culture plate at a density of 5 × 10^3^ cells per well and incubated for 24 h. Subsequently, the cells were treated with varying concentrations of PCA, free ETS and ETS@PCA. After incubation for 24 hours, the culture medium in the cell wells was replaced by the fresh medium containing CCK-8 (10%) and incubated for 2 hours. The absorbances of each well were measured at 450 nm on a microplate reader. The relative cell viability (%) was determined by comparing the absorbance values of sample wells with those control wells.

### *In vitro* cell apoptosis

4T1 cells (2 × 10^5^ cells per well) were seeded in a 6-well plate for 24 h. After incubation with PBS, PCA, ETS and ETS@PCA (ETS concentration: 2 μM) for 24 h, both floating and adherent cells were collected, washed with PBS, and stained with annexin V-fluorescein isothiocyanate (FITC) and propidium iodide (PI). The cells were further incubated for 15 min in the dark. The percentage of apoptotic cells was quantified by flow cytometry.

### *In vivo* antitumor efficiency

The female BALB/c mice aged 5–6 weeks were subcutaneously injected with 4T1 cells (1 × 10^6^) into their abdomen. When the tumor volume of the mice reached 80 mm^3^ approximately, the 4T1-bearing mice were randomly divided into four groups (*n* = 5) and treated with saline, ETS (5 mg kg^−1^), ETS@PCA (an equivalent dosage of ETS 5 mg kg^−1^) and PCA (an equivalent dosage of ETS@PCA) *via* intravenous administration through the tail. All mice were treated every two days for a total of 12 days. Tumor volumes and body weights were recorded every other day. The day when the treatment started was designated as day 0. Tumor volume was measured using a caliper, and the calculation was as follows: tumor volume (*V*) = *a* × *b*^2^/2, where *a* represents the major axis and *b* represents the minor axis of the tumor. The relative tumor volume was calculated according to the equation: *V*/*V*_0_ (*V*_0_ is the initial tumor volume before the start of treatment). All the mice were sacrificed on day 20. The major organs (heart, liver, lung and kidney) of various groups were harvested to evaluate systemic toxicity by histological H&E staining. TUNEL staining was performed on tumor sections to detect apoptotic cells, followed by observation using CLSM.

## Results and discussion

### Preparation and characterization of ETS@PCA

**Scheme 2:**
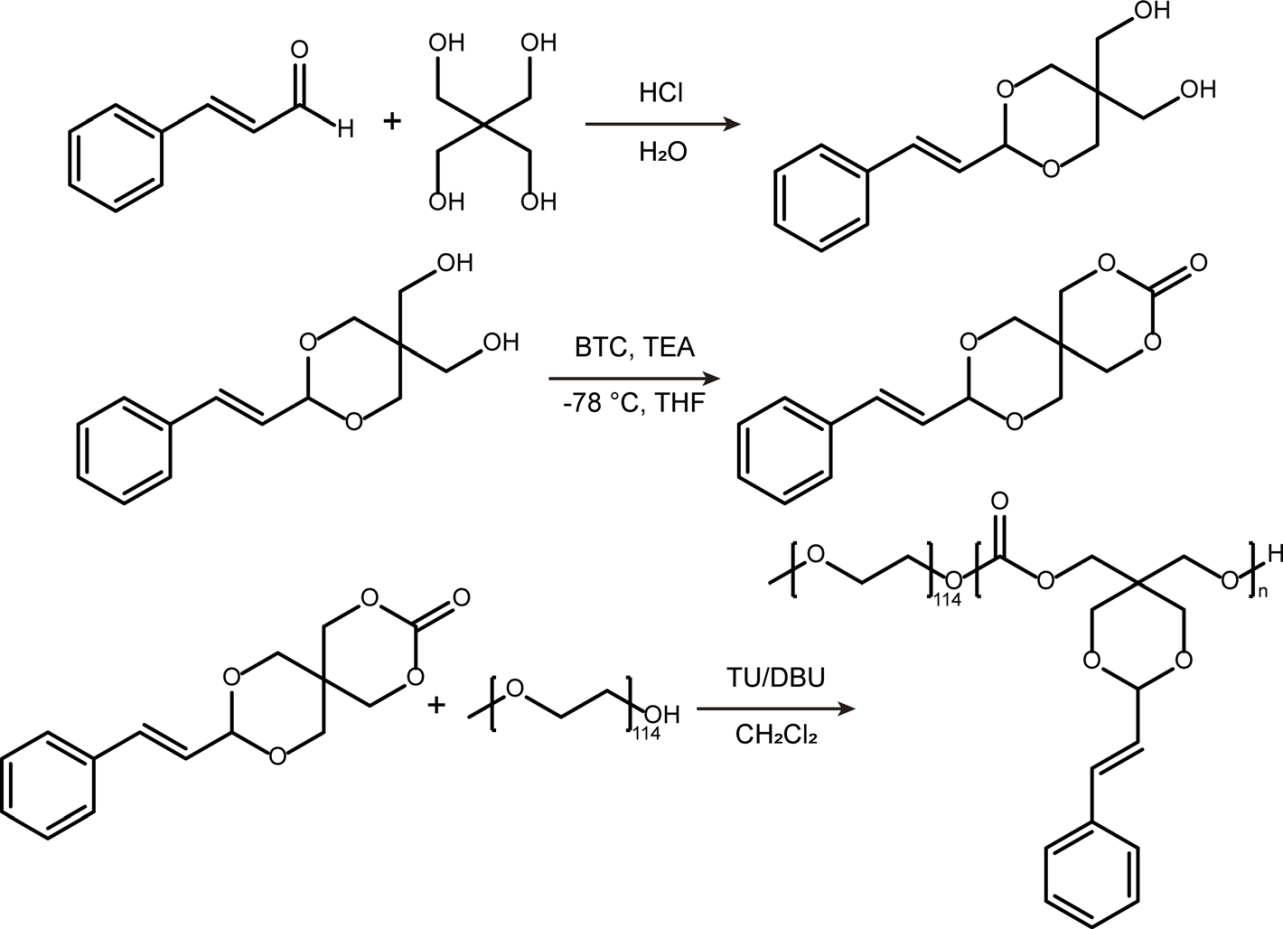
Synthesis process of PEG-PCA.

Scheme 2 depicts the synthetic pathway of an amphiphilic polymer PEG-PCA. Initially, a condensation reaction occurred between CA and pentaerythritol in an aqueous medium, resulting in the formation of acetalized CA (PERCA). Subsequently, PERCA underwent a ring-closing reaction *via* triphosgene under low-temperature conditions, yielding CA-functionalized cyclic carbonate compounds (STO). Lastly, the ring-opening polymerization (ROP) of STO was carried out using mPEG_5000_ as the initiator and a combination of DBU and TU as catalysts, leading to the formation of an amphiphilic polymer PEG-PCA. The chemical structures of PERCA and STO were confirmed using proton nuclear magnetic resonance spectroscopy (^1^H NMR). The peak at 5.03 ppm in the ^1^H NMR spectrum (Fig. S1A†) corresponds to the characteristic acetal proton signal. Additionally, the integral ratios corroborate a 1 : 1 stoichiometry between CA and pentaerythritol moieties, further supporting the successful synthesis of PERCA. A comparison of the ^1^H NMR spectra of STO (Fig. S1B†) and PERCA revealed a downfield shift of the methylene protons adjacent to the hydroxyl group from 3.60 and 3.21 ppm to 4.65 and 4.05 ppm. This shift is attributed to the formation of six-membered cyclic carbonate structures. ^13^C NMR spectroscopy further corroborated the structural transformation. As shown in Fig. S2,† the carbon signals at 61.50 and 59.95 ppm, corresponding to the terminal –CH_2_OH group in PERCA, exhibited downfield shifts to 71.37 and 70.31 ppm, while a new peak at 147.90 ppm emerged, consistent with the formation of the carbonate (–OCOO–) group in STO.

**Fig. 1:**
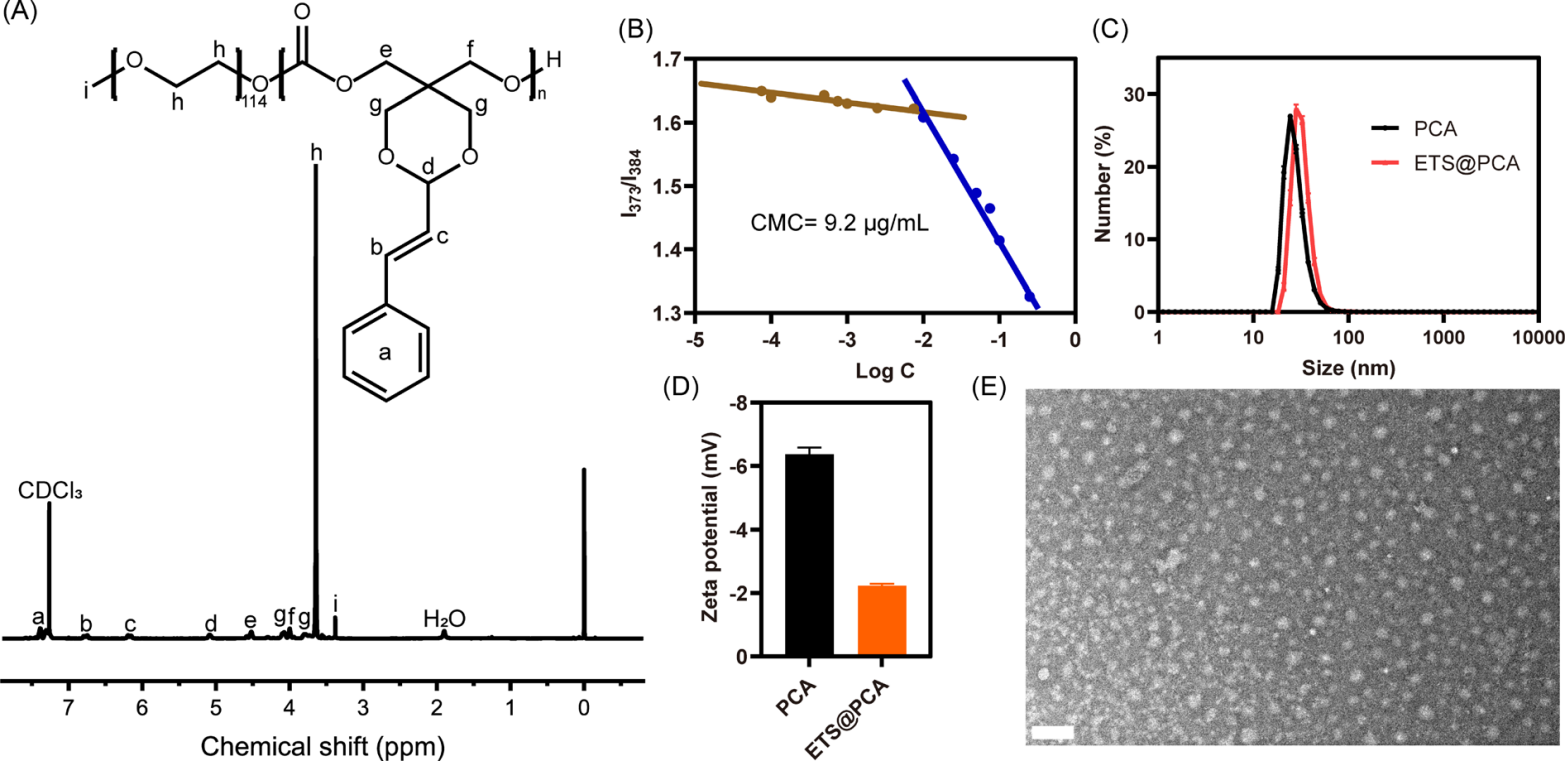
Physicochemical characterization of PEG-PCA. (A) 1H NMR spectrum of PEG-PCA. (B) Critical micelle concentration of PEG-PCA in water. (C) Hydrodynamic size and (D) zeta potential of PCA and ETS@PCA. (E) TEM image of ETS@PCA. Scale bar: 100 nm.

The ^1^H NMR spectrum in Fig. 1A depicts the PEG-PCA copolymer, exhibiting distinct signals for the PEG segment (*δ* 3.64 ppm) and the polycarbonate main chain segment (*δ* 4.09, 3.79 ppm). Comparing the ^1^H NMR spectra of PEG-PCA and STO revealed no significant changes in the acetalized CA section. The average degree of polymerization (DP) of PEG-PCA was determined to be approximately 13 by comparing the integration intensities of the acetal protons (peak d) and the methylene protons of PEG (peak h) in the ^1^H NMR spectrum. FTIR analysis was performed on both the cyclic monomer and PEG-PCA. For monomer STO, the characteristic carbonate peak was found at 1765 cm^−1^. After polymerization, the carbonate peak of PEG-PCA moved to 1749 cm^−1^. Notably, the peak intensity of polymer at 2980 cm^−1^, assigned to the C–H stretching vibration of the PEG moiety, was significantly enhanced compared to the carbonate peak. (Fig. S3†) These findings are consistent with previous observations. The gel permeation chromatography (GPC) curve (Fig. S4†) exhibited an elution time of 26.2 min, with a number-average molecular weight (Mn) of 8500 g mol^−1^ and a polydispersity index of 1.27, confirming the controlled synthesis of the PEG-PCA copolymer. Moreover, we studied the thermal behavior of PEG-PCA by TGA and DSC, respectively. As shown in Fig. S5A,† there was no significant degradation of PEG-PCA below 200 °C. On the other hand, PEG-PCA was found to have two *T*_m_ values at 42.2 °C and 49.3 °C, which might belong to polycarbonate and PEG segments, respectively (Fig. S5B†). The above results imply that the polymer as a drug carrier possesses well thermal stability and can maintain a solid state at physiological temperature.

The amphiphilic polymer PEG-PCA self-assembles in aqueous media to form core–shell micelles (denoted as PCA). This micellar structure facilitates the encapsulation of the lipophilic anticancer drug ETS through the dialysis method (denoted as ETS@PCA). The critical micelle concentration (CMC) was determined using pyrene as a fluorescence probe. As illustrated in Fig. 1B, the CMC value of PEG-PCA was approximately 9.2 μg mL^−1^. The low CMC value contributes to enhanced micelle stability and prolonged blood circulation time. The hydrodynamic diameter of PCA, measured by dynamic light scattering (DLS), was approximately 27.6 nm, and the zeta potential was approximately −6.36 mV. In comparison to the blank micelles, the particle size of ETS@PCA increased slightly to 32.3 nm, while the zeta potential decreased to −2.23 mV (Fig. 1C and D). The transmission electron microscopy (TEM) image in Fig. 1E revealed that ETS@PCA consisted of uniform spherical particles with a diameter of approximately 21 nm. This size is smaller than that measured by DLS, attributable to the dry state of nanoparticles during TEM analysis. TEM analysis revealed that PCA exhibited a marginally smaller particle size compared to the ETS@PCA, corroborating the findings obtained from the hydrate particle size measurements. (Fig. S6†) Nanoparticles within the size range of 10–200 nm can circumvent rapid renal clearance and evade capture by the reticuloendothelial system. They also demonstrate preferential accumulation within tumor tissues due to their localization within the tumor capillary plexus and reduced lymphatic drainage, resulting in prolonged intratumoral residence time. Moreover, owing to the prevalence of negatively charged components on the inner surfaces of blood vessels and cells, the use of surface-negatively charged nanoparticles can minimize non-specific adsorption to non-tumor tissues during *in vivo* transport, thereby reducing losses.^23^

### Drug release and cellular uptake

**Fig. 2:**
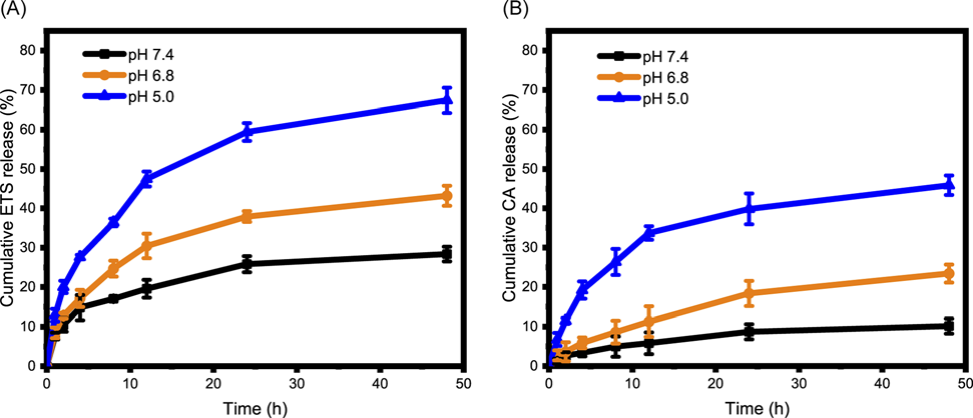
Acid-responsive release of ETS and CA. *In vitro* ETS (A) and CA (B) release profiles of ETS@PCA in PBS containing 0.5% (w/v) Tween 80 at pH 7.4, 6.8, and 5.0 (*n* = 3).

The encapsulation of ETS within ETS@PCA nanoparticles was quantified using HPLC, revealing a favorable drug loading capacity of 8.46 ± 0.14% and encapsulation efficiency of 87.9 ± 1.5%. This indicates the efficient drug-carrying capability of the PEG-PCA system. The pH-dependent release of cargo (CA and ETS) from ETS@PCA was studied using a dialysis method. Mimicking physiological environments of normal tissues (pH 7.4), tumor tissues (pH 6.8), and lysosomes in cells (pH 5.0),^24^ ETS@PCA nanoparticles were incubated in PBS at these corresponding pH values. The cumulative release of ETS reached 59% and 67% at pH 5.0 after 24 and 48 hours, respectively (Fig. 2A). Conversely, ETS@PCA displayed lower ETS release in PBS (pH 7.4), suggesting good nanoparticle stability under physiological conditions. Fig. 2B illustrates that the release rate of CA gradually increased as the pH decreased from 7.4 to 5.0, which can be attributed to the accelerated hydrolysis of acetal bonds under acidic conditions. Furthermore, this conclusion was supported by comparing the ^1^H NMR spectra of PEG-PCA before and after the hydrolysis of acetal bonds (Fig. S7†). These results indicate that the designed pH-responsive micelles are capable of mediating the controlled release of drugs upon exposure to acidic environments, thereby enhancing the antitumor efficacy.

**Fig. 3:**
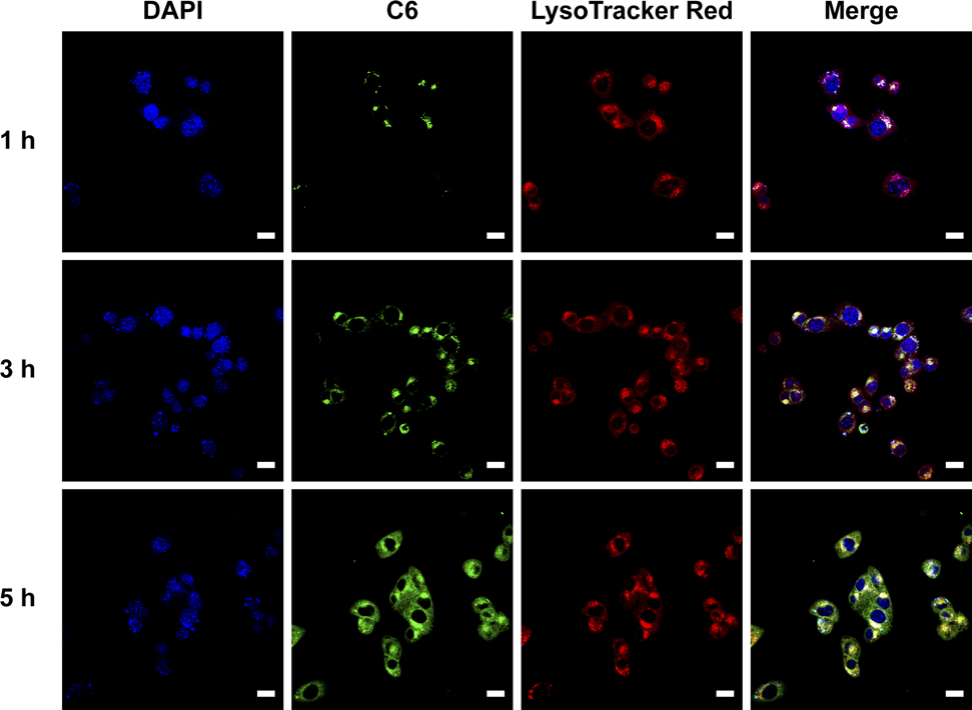
CLSM images of coumarin 6-loaded nanoparticles (C6@PCA) (green) in 4T1 cells after different incubation times. Nuclei were stained by DAPI (blue); *endo*/lysosomes were stained by LysoTracker Red (red). Scale bar: 20 μm.

Coumarin 6 (C6), a fluorescent probe, is widely used to monitor nanoparticle-based drug delivery *via* fluorescence confocal microscopy, as cellular internalization is critical for drug efficacy.^25^ To investigate the intracellular distribution of nanoparticles, C6@PCA was prepared through dialysis, with a C6 loading of approximately 0.5% (w/w) in the nanoparticles. Cellular localization was evaluated using laser confocal microscopy after incubating 4T1 cells with C6@PCA for 1, 3, and 5 hours, respectively. As shown in Fig. 3, intense green fluorescence from C6 was observed within the cells, and the fluorescence intensity increased with longer incubation times (Fig. S8†). Moreover, at 1 h, there was an obvious overlap between the green fluorescence of C6 and red fluorescence of LysoTracker probes in 4T1 cells, which indicated that the C6@PCA micelles were located in *endo*/lysosomes. With the increase of time from 1 h to 3 h, the yellow fluorescence was further enhanced. In contrast, at 5 h, yellow fluorescence weakened and green fluorescence of C6 emerged in the cytoplasm, indicating the release of the cargo in the micelles. These results demonstrate the successful and efficient uptake of C6@PCA in the 4T1 cells.

### *In vitro* GSH depletion and ROS elevation

**Fig. 4:**
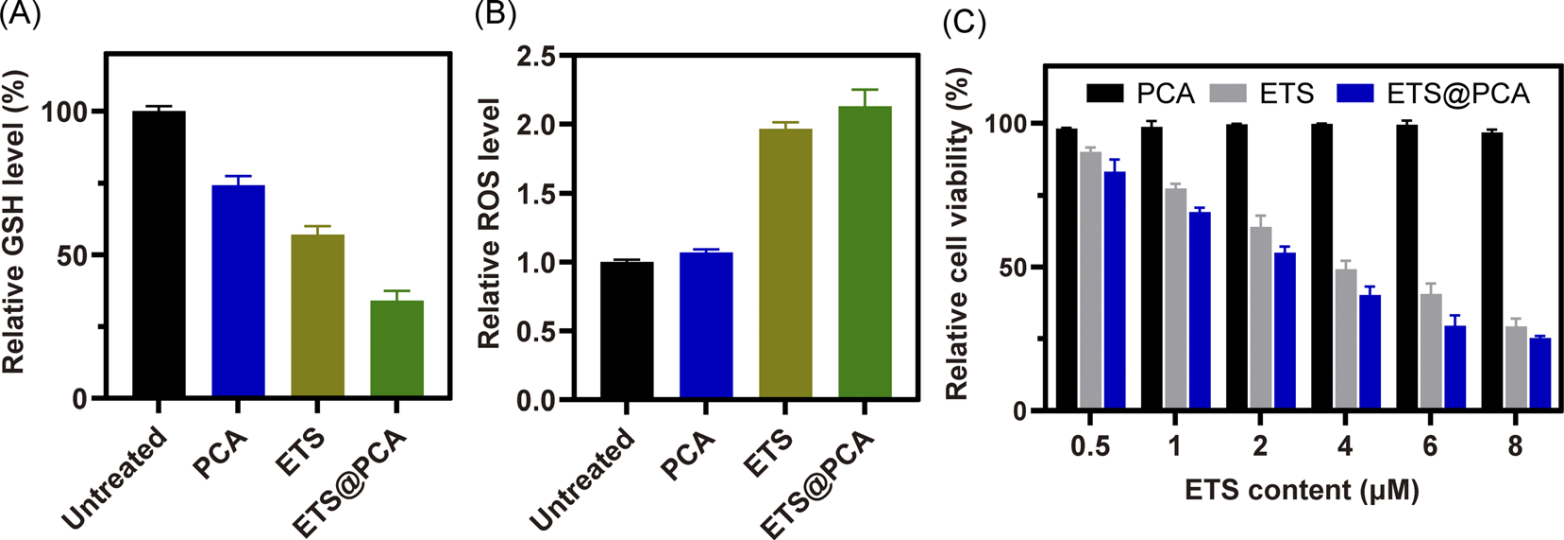
*In vitro* analysis of GSH (A) and ROS (B) level in 4T1 cells treated with PCA, ETS and ETS@PCA, respectively, for 24 h. (C) *In vitro* cytotoxicity evaluation of 4T1 cells after treatment with various doses of PCA, ETS and ETS@PCA for 24 hours, respectively.

GSH effectively protects cells from the cytotoxic effects of ETS by preventing their metabolism and promoting their efflux.^26,27^ To assess the downregulation of GSH by our designed polymer-loaded nanoparticles (ETS@PCA), 4T1 breast cancer cells were exposed to free ETS, PCA, and ETS@PCA, respectively. Subsequently, we measured the alterations in intracellular GSH and ROS levels, with untreated cells serving as controls. Fig. 4A demonstrates that exposure to free ETS alone led to an approximate 43% reduction in intracellular GSH levels in 4T1 cells, whereas blank micelle PCA also caused a decrease, albeit less pronounced (around 25%). Notably, ETS@PCA treatment resulted in the most substantial GSH depletion, with levels reaching approximately one-third of controls. Furthermore, as shown in Fig. 4B, ETS@PCA treatment significantly increased intracellular ROS levels in 4T1 cells, more than doubling those observed in the control group. These findings clearly indicate that ETS@PCA effectively disrupted the redox homeostasis in 4T1 cells, leading to a rapid depletion of GSH levels, which has the potential to enhance the cytotoxicity of ETS.

### *In vitro* anti-proliferative assay

To evaluate the pharmacological activity of ETS@PCA, the survival rate of 4T1 cells was investigated after treatment with different formulations using CCK-8 assay. As depicted in Fig. 4C, the blank micelles exhibited minimal cytotoxicity at all concentrations, indicating good biocompatibility of the PCA carrier, a key prerequisite for drug delivery systems.^28^ The data also suggest that GSH depletion alone might be inadequate for inducing cancer cell death. Encouragingly, the viability of 4T1 cells in ETS@PCA groups significantly decreased with increasing ETS concentration. ETS exhibited an IC_50_ (half maximal inhibitory concentration) of 3.58 μM, whereas ETS@PCA demonstrated a lower IC_50_ of 2.45 μM after 24 hours of treatment, suggesting enhanced antiproliferative activity against 4T1 cells. This potentiation likely arises from the combination of released CA and ETS, which aligns with our previous findings on GSH depletion.

**Fig. 5:**
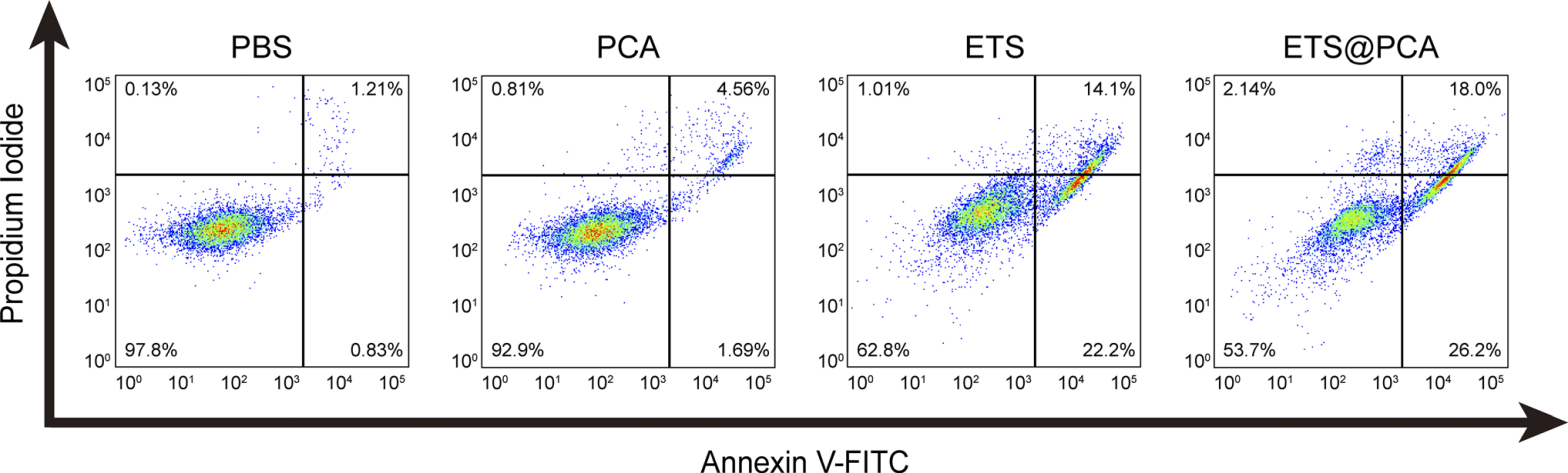
Flow cytometry analysis of 4T1 cell apoptosis induced by ETS@PCA using annexin V-FITC/PI staining. The lower right and upper right quadrants represent early and late apoptotic cells, respectively.

To determine the apoptotic effects, 4T1 cells were exposed to PCA, ETS, and ETS@PCA for 24 hours. Annexin V-FITC/PI staining followed by flow cytometry analysis was used to quantify apoptosis (Fig. 5). Untreated cells were included as controls. The PCA group exhibited a marginal increase in the number of apoptotic cells compared to the control group. In the ETS group, the apoptosis rate was 36.3%. Interestingly, at the same dose, ETS@PCA treatment resulted in a higher apoptosis rate of 44.2% in 4T1 cells. These findings provide further evidence that PCA enhances the cytotoxic effect of ETS on tumor cells.

### *In vivo* evaluation of ETS@PCA

**Fig. 6:**
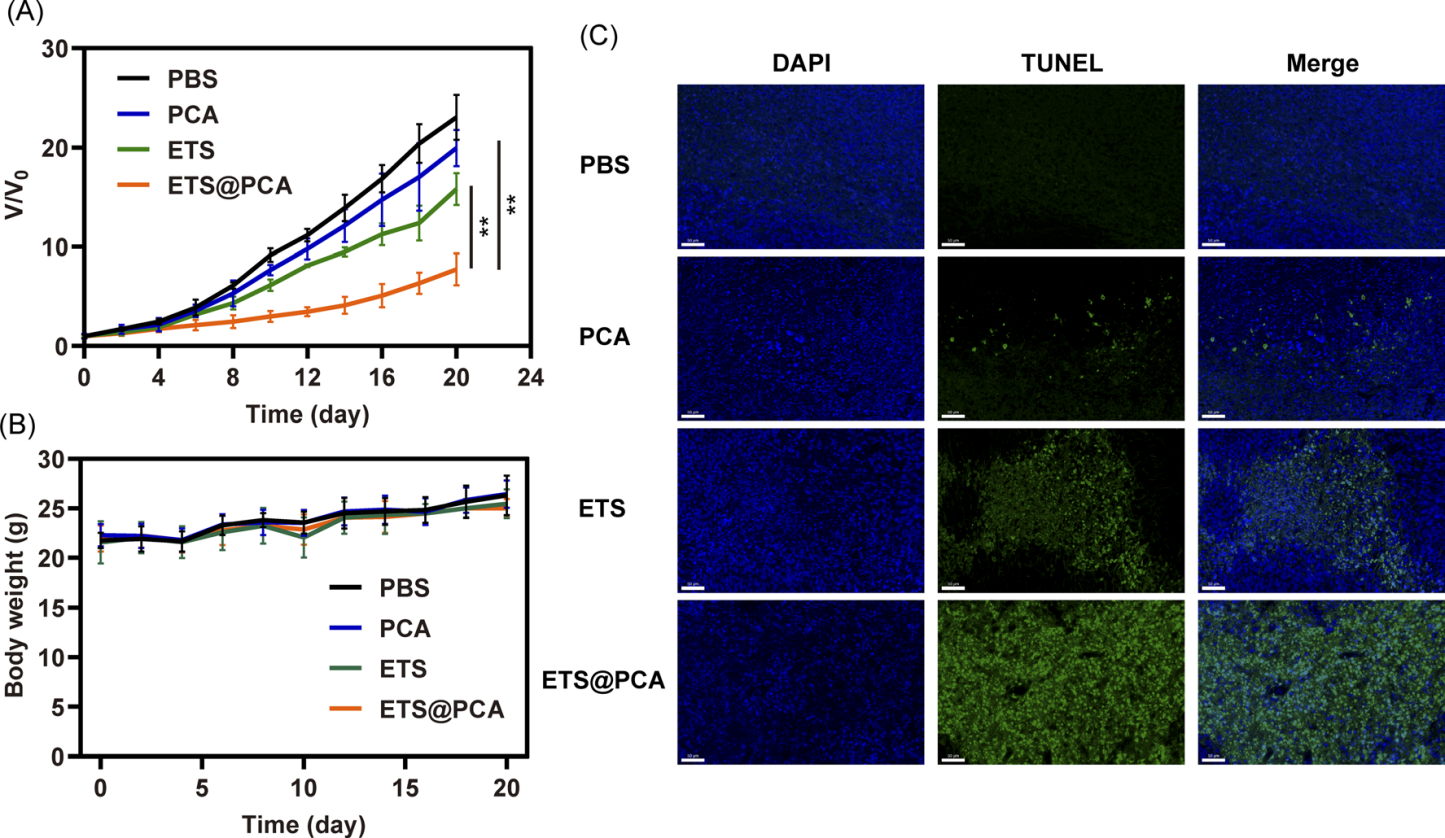
*In vivo* anticancer efficacy of ETS@PCA in 4T1 tumor-bearing xenograft mice. (A) Tumor growth-inhibitory (B) body weight changes during treatment. (C) TUNEL staining of tumor sections after treatment (scale bar: 50 μm). (**p < 0.01).

To evaluate the antitumor efficacy of ETS@PCA *in vivo*, a mouse model bearing 4T1 tumors was employed. Mice received intravenous injections of PBS, free ETS, or ETS@PCA (5 mg kg^−1^ ETS) on days 0, 3, 6, 9, and 12. Tumor volume and body weight were monitored for 20 days. Fig. 6A revealed minimal tumor growth inhibition by PCA compared to the PBS control. The free ETS group exhibited moderate inhibition, whereas mice treated with ETS@PCA displayed significantly stronger tumor suppression (*p* < 0.01). This enhanced antitumor efficacy is likely due to increased drug accumulation at the tumor site, facilitated cellular uptake of the nanomedicine, and the promotive effect of CA on ETS. Analysis of body weight (Fig. 6B) revealed no significant differences among the experimental groups, suggesting minimal systemic toxicity associated with ETS@PCA administration. This observation was further corroborated by histological examination of heart, kidney, liver, and lung tissues, which showed no appreciable damage (Fig. S9†). The low inherent toxicity of the raw materials used to synthesize PEG-PCA likely contributes to its safety profile. Following the hydrolysis of the fragile sites in PEG-PCA, such as acetal and carbonate bonds, within the body, the constituent PEG and pentaerythritol are eliminated through established metabolic pathways.

To assess the pro-apoptotic activity of ETS@PCA, a TUNEL assay was performed on tumor sections (Fig. 6C). Green fluorescence intensity progressively increased from PBS, PCA, and ETS groups to the ETS@PCA group, demonstrating potent pro-apoptotic activity in the latter, consistent with *in vitro* findings.

## Conclusions

In this study, we synthesized an amphiphilic acid-responsive polycarbonate, PEG-PCA, *via* a three-step reaction employing biocompatible materials. Leveraging the GSH-depleting ability of CA, PEG-PCA was employed as a carrier to enhance the therapeutic potency of ETS in cancer cells by overcoming its detoxification mechanisms. *In vitro* experiments demonstrated that ETS@PCA released CA in lysosomes of tumor cells, effectively depleting intracellular GSH levels and potentiating the therapeutic effect of ETS. *In vivo* studies validated the superior tumor suppression efficacy of ETS@PCA without causing significant systemic toxicity. In conclusion, this GSH-depleting nanomedicine represents a promising strategy to overcome the clinical limitations of ETS and enhance its therapeutic potential in cancer treatment.

## Ethical statements

BALB/c nude mice were obtained from the Hangzhou Ziyuan Experimental Animal Technology Co., Ltd All animal procedures were conducted in accordance with the recommendations of the Guide for the Care and Use of Laboratory Animals of the National Institutes of Health. This animal study was approved by the Ethics Committee of Yanxuan Biotechnology (Hangzhou) Co., Ltd (YXSW24013009810). Animal welfare was a primary concern, and all efforts were made to minimize suffering.

## Author contributions

The manuscript was written with contributions from all authors. All authors have approved the final version of the manuscript.

## Conflicts of interest

The authors declare that they have no competing interests.

## Supplementary Material

RA-014-D4RA02468K-s001
