# Supplementary material for: Facile synthesis of an acid-responsive cinnamaldehyde-pendant polycarbonate for enhancing the anticancer efficacy of etoposide via glutathione depletion
Source: RSC Adv. 2024 May 13;14(22):15365–73. doi: 10.1039/d4ra02468k (PMC11089533; doi:10.1039/d4ra02468k)
Supplement: RA-014-D4RA02468K-s001 [file RA-014-D4RA02468K-s001.pdf]

Electronic Supplementary Material (ESI) for RSC Advances.
This journal is © The Royal Society of Chemistry 2024

Electronic Supplementary Information

Facile Synthesis of an Acid-Responsive Cinnamaldehyde-Pendant Polycarbonate for Enhancing the Anticancer Efficacy of Etoposide via Glutathione Depletion

*Shaojie Wu, Kuofei Liao, Jiamin Chen and Feng Li**

Key Laboratory of Biomedical Polymers of Ministry of Education, College of Chemistry and Molecular Science, Wuhan University, Wuhan, 430072, PR China

E-mail address: lifeng@whu.edu.cn (F. Li)

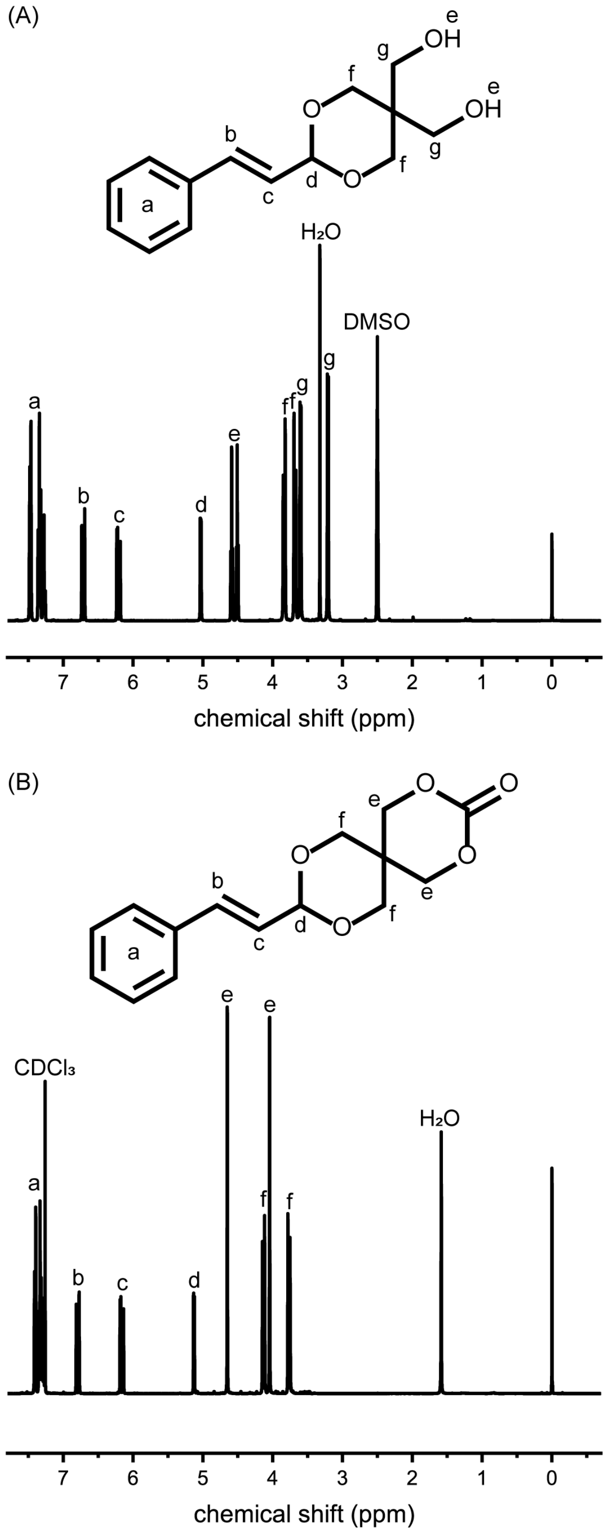

Figure S1. (A) ^{1}H NMR spectrum of PERCA. (B) ^{1}H NMR spectrum of STO.

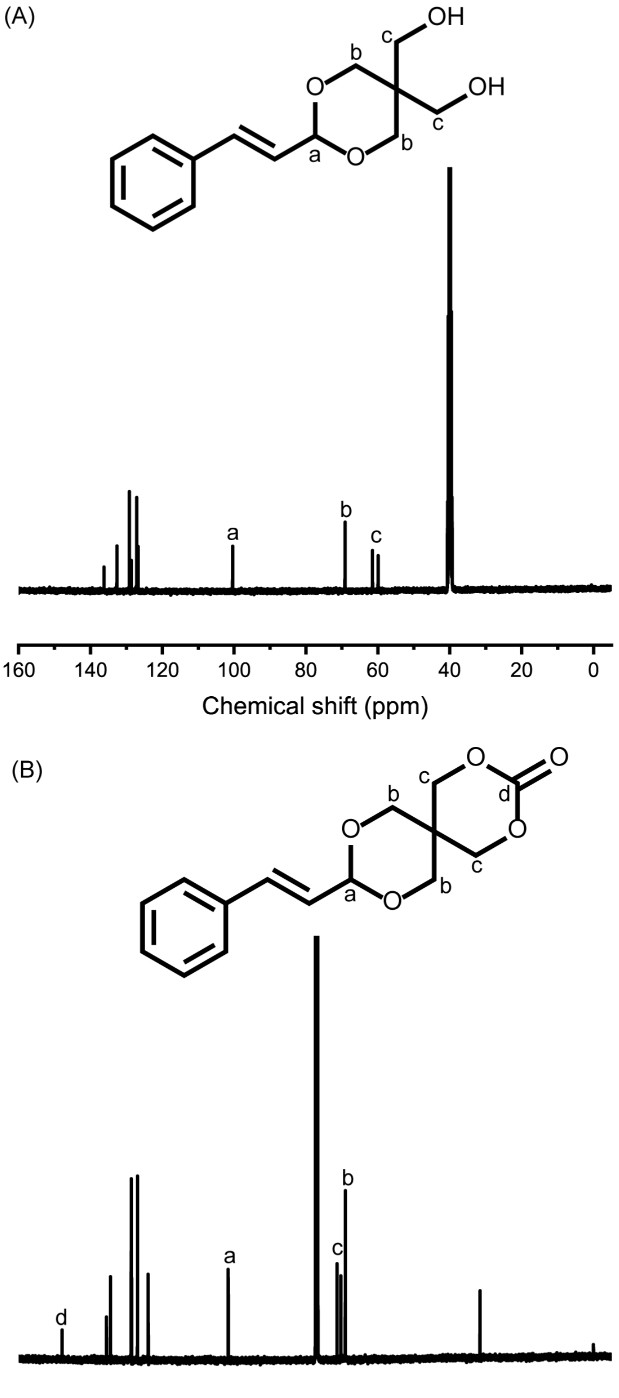

Figure S2. (A) ^{13}C NMR spectrum of PERCA. (B) ^{13}C NMR spectrum of STO.

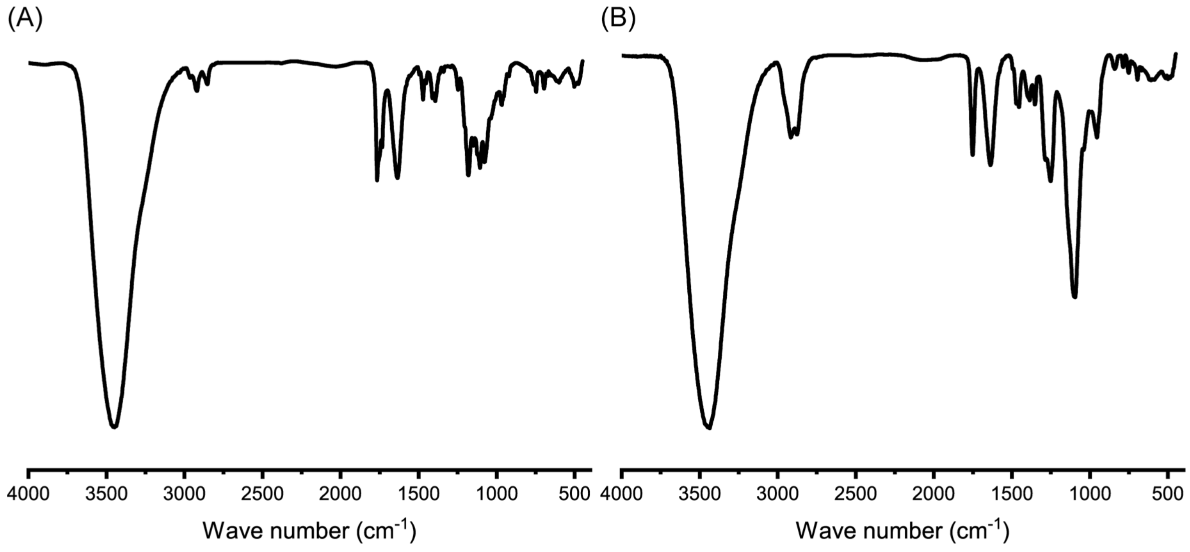

Figure S3. FTIR spectra of STO (A) and PEG-PCA (B).

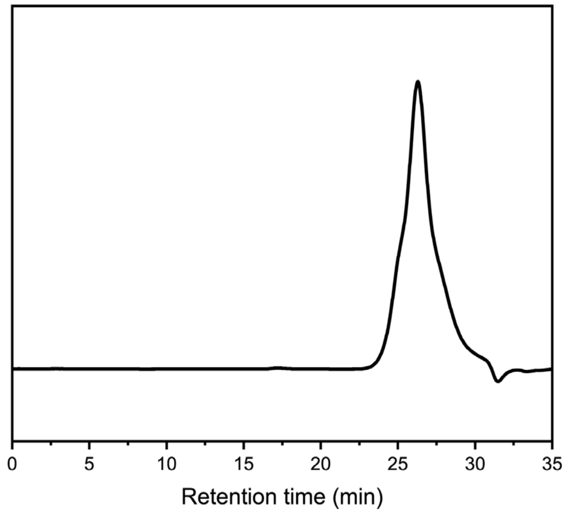

Figure S4. GPC curve of PEG-PCA.

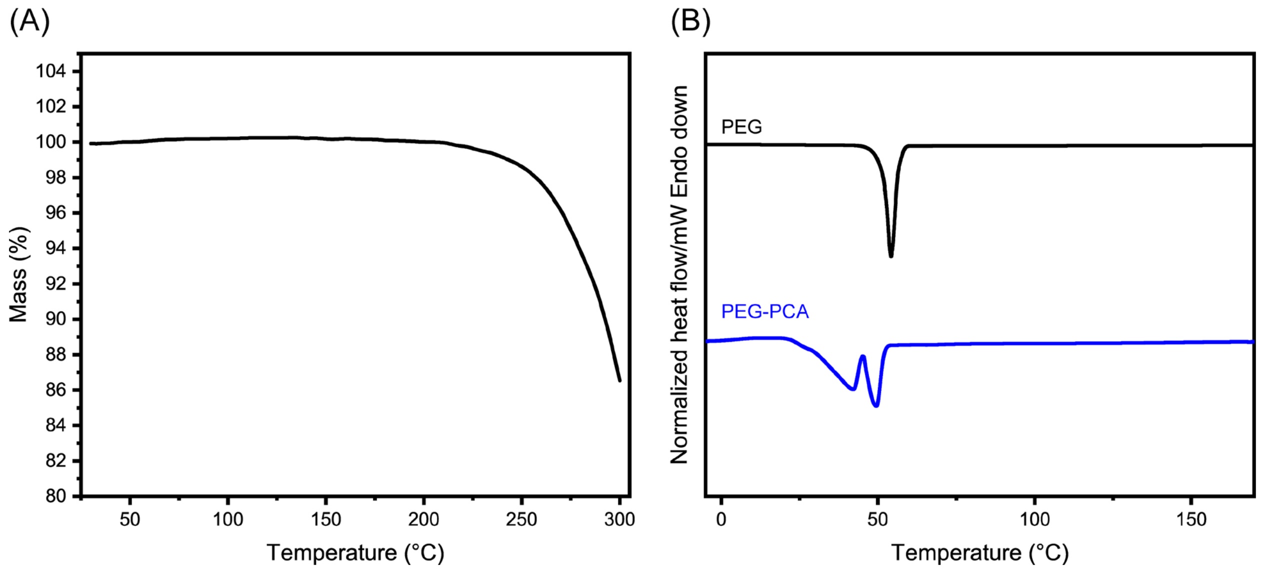

Figure S5. TGA curve (A) and DSC curve (B) of PEG-PCA.

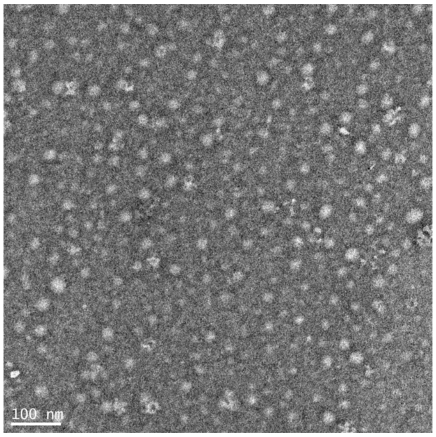

Figure S6. TEM image of PCA. Scale bar: 100 nm.

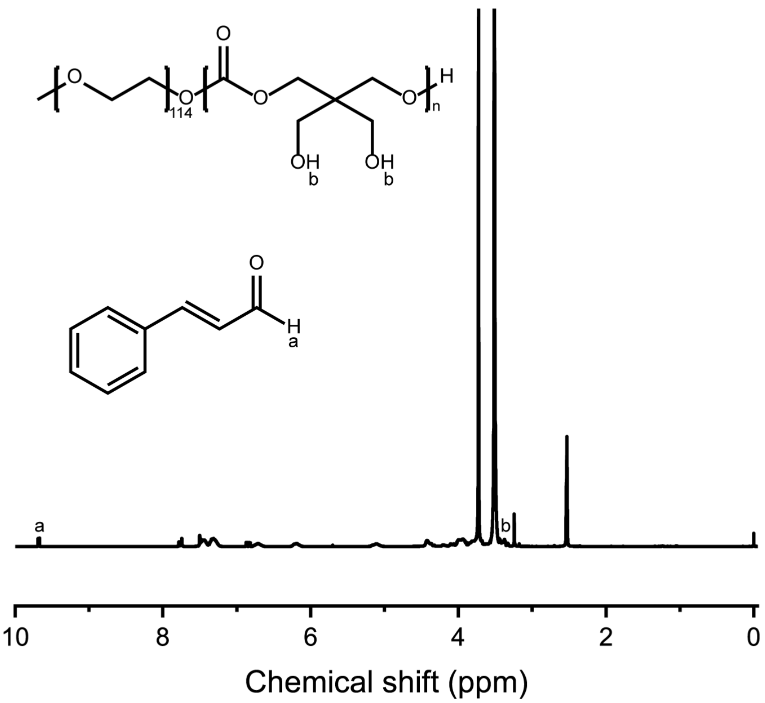

Figure S7. ^{1}H NMR spectrum (D_2O (pD = 4.0)/DMSO-d_6, 400 MHz) of PEG-PCA after hydrolysis.

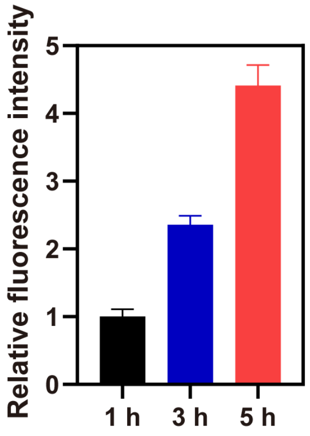

Figure S8. Quantitative analysis of C6 fluorescence intensity over time (n = 3).

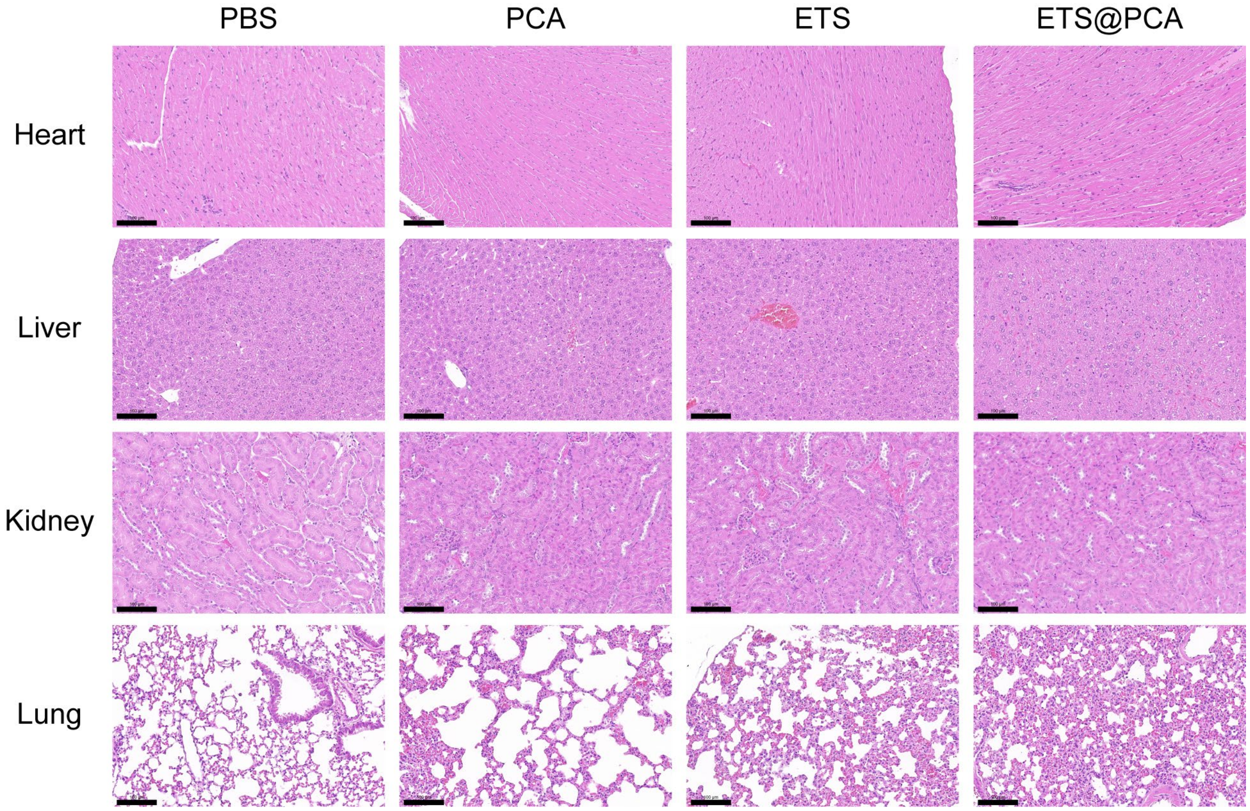

Figure S9. Histological analysis of major healthy organs (heart, kidney, liver, lung) by H&E staining (scale: 100 μm).
